# Therapeutic Pearls at Random Strung

**Published:** 1881-09

**Authors:** 


### THERAPEUTIC PEARLS AT RANDOM STRUNG.

### Impotence in the Male.

Bathe the testicles in pepper tea, or dilute tincture of capsicum once a day, and inject twenty grains of borax, dissolved in a small quantity of water, into the rectum with a small syringe. The injection may be repeated every night until relief is obtained.

### Pruritus (Itching) of the Anus.

*Sulphurous acid*, diluted at first, and sopped on the surface around the anus two or three times per day, will, within a week, destroy the parasitic plant which gives rise to the disease.

### Pruritus of the Vulva.

This most distressing affection may generally be relieved by a few applications of the following formulæ. The parts should be bathed well with cold water and castile soap before using the medicines. Apply ℞ No. 1 freely, and in a few minutes apply ℞ No. 2, to any abrasions or ulcers which may exist, by sprinkling them well with it.

No. 1.

| | | | |
|---|---|---|---|
| ℞ | Pulv. Borax, . . . . . . | ℥ ss. | |
| | Sulph. Morphia, . . . . | grs. x. | |
| | Rose Water, . . . . . . | ℥ iij. | M. |

No. 2.

| | | |
|---|---|---|
| ℞ | Prepared carbonate zinc, . . . | ℥ i. |
| | (Finely powdered.) | |

The following Ointment applied several times per day will often afford prompt relief in *Pruritus*, viz.:

| | | | |
|---|---|---|---|
| ℞ | Calomel, . . . . . . . | ʒ ij. | |
| | Pulv. Camphor, . . . . . | ʒ ss. | |
| | Sulph. Morphia, . . . . . . | ℈ i. | |
| | Acetate Lead, . . . . . . | ʒ ss. | |
| | Simple Cerate, . . . . . . | ℥ ss. | M. |

Bathe the vulva thoroughly with tepid water, and wash twice a day with a solution containing *half a drachm* of SULPHO-CARBOLATE of ZINC, to an *ounce* of DISTILLED WATER. Allow the parts to dry without wiping.

FOR THE SAME.

| | | |
|---|---|---|
| ℞ | Bichloride of mercury, . . . | 1 part. |
| | Alum, . . . . . . . . | 20 parts. |
| | Starch, . . . . . . . | 100 parts. |
| | Water, . . . . . . | 2500 parts. |

Mix and apply freely to the parts.

FOR THE SAME.

Topical applications of *cider vinegar* was strongly recommended by the late Professor Samuel Henry Dickson in this troublesome affection.

FOR THE SAME.

| | | | |
|---|---|---|---|
| ℞ | Pulv. gum camphor. | | |
| | Hydrate chloral, . . . . . | āā ʒ i. | |
| Rub together when liquid, add | | | |
| | Sing. Aquæ Rosæ, . . . . . | ℥ i. | M. |

S. Apply to the parts affected, but not when the skin is broken.

FOR THE SAME.

| | | | |
|---|---|---|---|
| ℞ | Ol. erigeron. | | |
| | Tinct. canthar. | | |
| | Tinct. aconite, rad., . . . . | āā f. ʒ ss. | |
| | Alcohol, . . . . . . . . | ʒ ij. | |
| | Syrup Acac., . . . . . . | ℥ ij. | M. |

S. A teaspoonful every two or three hours, and use as required locally recipes Nos. 1 and 2, preceding.

FOR THE SAME.

Dr. Tauzsky recommends the following (*New York Medical Record*, vol. ii, 1880, p. 387):

℞ Pulvis acaciæ, . . . . . ʒ ii.

Bals. Peruvian, . . . . . . ʒ i.

Ol. amygdalæ, . . . . . . ʒ iss.

Aquæ rosæ, . . . . . . ℥ i. M.

Apply freely with a camel's hair brush eight or ten times a day to the itching part.

FOR THE SAME.

℞ Carbolic acid (full strength) . . grs. xv.
Glycerine and rose water, . . . āā ℥ ij. M.

Apply locally several times a day.

OR,

℞ Carbolic acid, . . . . . . ʒ i.

Hyposulphite soda, . . . . . ʒ ij.

(Or Pulv. Borax.)

Glycerine, . . . . . . . ℥ ij.

Water, . . . . . . . ℥ vi.

Mix and apply by saturating cloth to the part or using syringe, as necessary.

